# Eye bank prepared versus surgeon cut endothelial graft tissue for Descemet membrane endothelial keratoplasty: An observational study

**DOI:** 10.1097/MD.0000000000006885

**Published:** 2017-05-12

**Authors:** Marie Regnier, Céline Auxenfans, Delphine Maucort-Boulch, Anne-Sophie Marty, Odile Damour, Carole Burillon, Viridiana Kocaba

**Affiliations:** a Service d’ophtalmologie; b Banque de Tissus et de Cellules des Hospices Civils de Lyon, Hôpital Edouard Herriot, Lyon; c Université Claude-Bernard Lyon-I, Villeurbanne; d Service de Biostatistique, Hospices Civils de Lyon, Lyon; e CNRS UMR5558, Laboratoire de Biométrie et Biologie Evolutive, Equipe Biostatistique-Santé, Villeurbanne, France.

**Keywords:** Descemet membrane endothelial keratoplasty, endothelial graft preparation, eye bank, Fuchs endothelial corneal dystrophy

## Abstract

The purpose of this article is to examine outcomes of Descemet membrane endothelial keratoplasty (DMEK) performed with cornea bank (CB) prestripped tissue and surgeon stripped tissue (SST).

This retrospective study examined subjects who underwent DMEK with CB or surgeon prepared tissue for Fuchs endothelial corneal dystrophy. Best-corrected visual acuity (BCVA), corneal thickness, endothelial cell count (ECC), and complications were examined before and throughout a 6-month postoperative period.

Eleven CB and 22 SST subjects were included. Six months after surgery, BCVA was 20/20 or better in 36.4% of CB and 22.7% of SST subjects (*P* = .43). Median logMAR BCVA was 0.10 (0.00–0.20, 20/25) in group CB and 0.10 (0.10–0.30, 20/25) in group SST. Median preoperative corneal thickness was 614.0 μm (577.5–662.0 μm) and 658.0 μm (606.0–689.0 μm) in CB and SST subjects, respectively (*P* = .37). Six months after surgery, median corneal thickness was lower in the CB group (571.0 μm [478.0–592.0 μm]), than in the SST group (576.0 μm [531.0–607.0 μm], *P* = .02). At 6 months, median ECC was 1500.0 cell/mm^2^ (1321.5–2049.0 cell/mm^2^, 41% decrease) in group CB and 1403.0 cell/mm^2^ (972.5–2010.7 cell/mm^2^, 46% decrease) in group SST (*P* = .70). Rebubbling was required in 5 CB (45.5%) and 15 SST (68.2%) subjects (*P* = .39).

Fuchs’ dystrophy patients have good anatomic and functional DMEK results. Similar outcomes and complication rates occurred with eye bank and surgeon prepared donor tissue.

## 1 Introduction

Descemet membrane endothelial keratoplasty (DMEK), first described by Melles et al^[1]^ in 2006, replaces only the damaged posterior cornea with careful removal and transplantation of Descemet membrane (DM) and the endothelium. This procedure allows for a faster and better visual recovery than a full-thickness corneal transplant.^[1–3]^ Before DMEK was developed, other endothelial graft techniques were in use, including deep lamellar endothelial keratoplasty (introduced in 1998)^[4]^ and Descemet stripping automated endothelial keratoplasty (DSAEK), both of which include replacing the endothelio-Descemet posterior stroma.^[5,6]^ Many surgeons are learning DMEK to treat corneal endothelium pathologies, including Fuchs endothelial corneal dystrophy (FECD),^[7]^ largely because this thin, regular graft does not include the stroma. This decreases hyperopic shift, astigmatism, higher order aberrations, and refractive changes following surgery compared with full-thickness transplants.^[8,9]^ Furthermore, only small incisions are needed for DMEK and suturing can be avoided. This reduces the risk of inflammation, graft rejection, and wound dehiscence.^[10]^ The efficacy of DMEK has been shown by examining long-term (5 years) visual recovery and outcome stability.^[11]^ However, the required manual dissection of donor corneas, the first step in performing DMEK, discourages many surgeons from learning the procedure. Donor tissue dissection can either be done in the operating room (OR, surgeon strip) or at an eye bank (technician strip).^[12]^ Learning to properly prepare donor tissue is challenging and requires long, time-consuming training for surgeons and eye bank technicians.^[13,14]^ In addition, when dissections are performed in the OR, endothelial quality control is not a possibility and mistakes made in tissue preparation can lead to procedure cancellations or postponements and subsequent hospital financial losses.^[15,16]^ For these reasons, most endothelial cell transplant procedures performed in the United States are done using tissue prepared by an eye bank.

Many scientific publications on DSAEK^[17–19]^ have confirmed equal endothelial cell graft quality between eye bank precut and OR surgeon-prepared tissue. However, while the precut technique has been evaluated previously,^[20,21]^ the comparison between tissue prepared by a trained eye bank technician and that prepared by a surgeon has not yet been studied for the DMEK procedure. Here, we examine endothelial graft outcomes following DMEK using cornea bank (CB) prepared tissue and surgeon stripped tissue (SST).

## 2 Methods

This study protocol was reviewed and approved by the Ethics Committee of Hospices Civils de Lyon (Lyon, France). All subjects provided written informed consent for use of their health information and all study conduct adhered to the tenets of the Declaration of Helsinki. In compliance with corneal donor contraindications, all corneas were obtained after verification that the donor was not registered in the National Register of Organ Donation Refusal and after obtaining consent from the donor's family.

### 2.1 Study participants

This retrospective, comparative study was conducted at Edouard Herriot Hospital (Lyon, France). Subjects were placed into the CB prestripped tissue group (conducted from May to December 2015) or the SST group (conducted from March 2014 to June 2015). Only FECD patients were included in the study. Subjects who had benefited from other corneal grafts and patients under 18 years old were excluded.

### 2.2 Donor tissue preparation

We obtained corneas as corneoscleral discs that had been excised from deceased donors within 24 hours of death. Corneas were then placed in transport medium (Stem Alpha 1, 7001 Stem Alpha, St Genis l’Argentière, France) and sent to the cornea bank. Upon receipt, the bank harvested and stored corneas in a preservation medium (Stem Alpha 2, 7002 Stem Alpha) at 31°C for up to 30 days. During storage, the following tests were performed: virology testing on donor blood (hepatitis B and C, HIV, human T-cell lymphotropic virus [HTLV] I, HTLV II, and syphilis), microbiological controls on storage medium, and evaluation of endothelial cell density (ECD, via cell counting) at the beginning and end of storage. Cell viability was also evaluated via cell numeration (under a microscope with a calibrated reticle after trypan blue staining). For donor corneal tissue to be sent for transplant, ECD had to be ≥ 2500 cells/mm^2^ with less than 2% of dead cells. Moreover, total cell loss during storage had to be less than 20%. Preservation medium allows survival of corneal endothelial cells. However, during storage, corneas become edematous and thicken. To reduce this edema and ensure that it did not interfere with surgery or make trephination more difficult, the cornea was transferred to a deswelling medium (Stem Alpha 3, 7003 Stem Alpha) between 24 and 96 hours before keratoplasty if tissue was to be dissected in the OR.

Tissue was either prestripped at the CB by 2 CB technicians or stripped in the OR by 2 trained surgeons (VK and A-SM). We chose to use the “no-touch” dissection technique with a surgical microscope under sterile conditions. The cornea was placed onto a silicone block and all uveal tissues were removed using a hockey stick blade. Descemet membrane was then detached around the tissue edge, beginning at the iris base, over a width of 2 mm. Trypan blue (0.06%; Croma-Pharma GmbH, Leobendorf, Austria) staining allowed for better visualization of DM, which was detached using DMEK forceps (MMSU1499 Kocaba DMEK forceps, Malosa Medical, Elland, England) from the periphery toward the center. This achieved separation of the entire DM. Correct detachment was controlled by buffered saline solution (BSS), and DM was trephined with an 8.0 mm diameter trephine. Due to the elastic properties of DM, the graft naturally rolls on itself into a double roll with the endothelium facing out. Following preparation, the graft was either placed into culture medium for conservation and delivery to the operating room within 2 days (CB-prepared tissue) or into BSS and trypan blue when grafted the same day (SST).

The ECD was assessed before dissection in both tissue groups and immediately after dissection in CB-prepared tissue. Previously validated CB methods for measuring ECD include manual cell counting by technicians using an inverted light microscope at a magnification of ×125 (Leica Leitz with a micrometric grid, Leica Microsystems, Buffalo Grove, IL) after coloration by trypan blue. Digital photographs of the corneal center were obtained using a Leica camera mounted on the microscope and anomalies (e.g., tears or cell death) were registered. Last, the correct DM position in the double roll was verified.

### 2.3 Surgical procedures

All DMEK were conducted under general, locoregional, or local anesthesia by 2 trained surgeons (VK and A-SM). Each patient received preoperative pilocarpine 1% or mydriaticum 0.5% and neosynephrine 10%, if a phacoemulsification was also being performed. The DMEK surgery was started by creating 4 paracenteses with a 15° blade (Beaver, Beaver-Visitec International, Waltham, MA) at 45°, 225°, 315°, and 135°.

An 8.5 mm descemetorhexis was performed using a reversed Sinskey hook (Moria, Antony, France) following injection of an air bubble into the anterior chamber to improve visibility. The principal incision was enlarged using a 2.2 mm blade at 135° (Alcon, Ft. Worth, TX). The air bubble was then removed to create a hypotonic eye. The graft was colored with trypan blue via two 3-minute soakings and sucked into a glass injector (single use cartridge G-38635, Geuder Laboratory, Bausch and Lomb). The injector was connected to a 5-mL syringe with BSS and the graft was injected through the main incision. The graft unfolded with gentle tapping on the cornea because of the 2 cannulas. There was no direct contact during the DMEK procedure between surgical instruments and the graft. Once the graft was unfolded and correctly positioned, the surgeon injected an air bubble into the anterior chamber to pin the graft against the stroma. All incisions were small enough to be self-sealing and suturing was not needed in any procedure. If phacoemulsification was also planned, it was performed immediately prior to DMEK using a viscoelastic cohesive that was entirely removed before graft injection. Each patient received a subconjunctival injection of 4 mg/mL betamethasone and a soft bandage contact lens to be worn for 3 days. Patients were also instructed to remain supine for 3 days. The postoperative eye drop regiment included 7 days of pilocarpine 1% 3 times a day, dexamethasone with monthly decreasing doses, and artificial tears for 1 year. If phacoemulsification had also been performed, a nonsteroidal anti-inflammatory agent (indomethacin) was prescribed for 1 month.

### 2.4 Data collection

All patients were examined before surgery and 7 days and 1, 3, and 6 months after surgery. At each examination, subjects underwent measurement of best-corrected visual acuity (BCVA), corneal thickness (Pentacam Oculus, Wetzlar, Germany), and endothelial cell count (ECC; specular microscopy [SP2000P, Topcon, Tokyo, Japan]). The air injection rate, dissection rate, graft failure rate, and complication rate were also noted.

### 2.5 Statistical analyses

Data are presented as a number (percentage) for categorical variables and median (1st–3rd quartiles) for continuous variables. The CB and SST group characteristics were compared using nonparametric tests (Wilcoxon test for continuous variables, *χ*^2^, or Fisher exact test for categorical variables). Changes over time were examined using the Friedman test, a nonparametric analysis of variance for repeated data. Statistical significance was defined as *P* < .05. All analyses were performed using R software (software 3.0.2, 2013, The R Foundation, Vienna, Austria).^[22]^

## 3 Results

### 3.1 Subject characteristics

Eleven subjects (8 females [72.7%]) received CB prepared tissue (CB group) and 22 subjects (17 females [77.3%]) received SST (SST group). There were no significant differences in subject characteristics between groups (Table 1). Median subject age was 73.0 years (65.5–80.0 years) in the CB group and 70.5 years (63.0–78.0 years) in the SST group (*P* = .60). The majority of subjects underwent a combined phacoemulsification/DMEK procedure (10 of 11 CB subjects [90.9%], 21 of 22 SST subjects [95.5%]; *P* > .99).

**Table 1:**
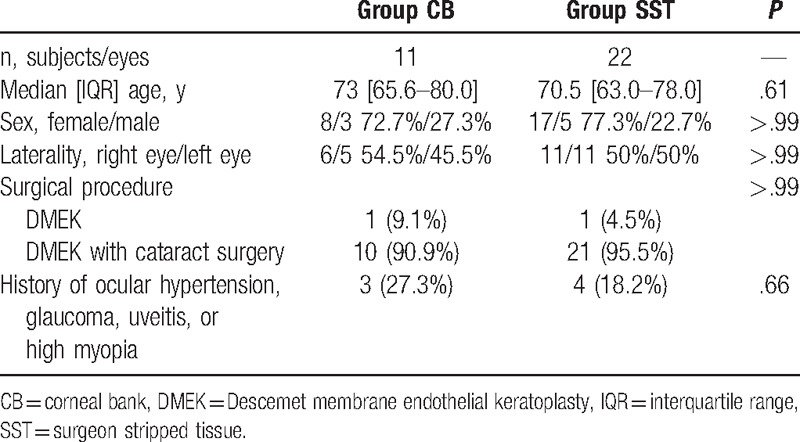
Subject characteristics and surgical procedures.

### 3.2 Donor and graft tissue characteristics

Median donor age was 62.0 years [57.0–69.5 years] in the CB group and 66.5 years [60.2–76.2 years] in the SST group, a slight difference that was not statistically significant (*P* = .24). Median postmortem time allowed for tissue collection was 6.0 hours (4.0–15.5 hours) in the CB group and 11.0 hours (7.5–18.5) in the SST group. Though fairly large, this difference was not statistically significant (*P* = .14). Median preservation time was 19.0 days (18.0–25.0 days) in the CB group and 15.5 days (13.0–21.5 days) in the SST group (*P* = .10). The 8 (72.7%) patients in the CB group were grafted at day 2; the 3 others were grafted at day 1. In contrast grafting was always performed on the same day as tissue preparation (in the OR) in the SST group. The median prestripped ECC was 2784 cells/mm^2^ (2612–2995 cells/mm^2^) in the CB group and 2694.5 cells/mm^2^**(**2594–2860 cells/mm^2^) in the SST group (*P* = .29). Prestripped ECC did not significantly differ between groups (Table 2). The median poststripped endothelial cell loss was 1% (0%–2%) in the CB group.

**Table 2:**
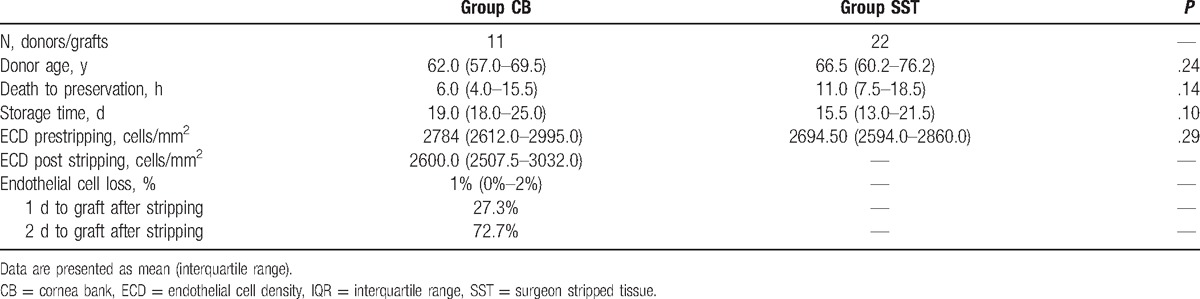
Donor and graft characteristics.

### 3.3 Visual outcomes

All subjects in the CB group and all but 1 subject in the SST group had a preoperative logMAR BCVA greater than 0.3 (worse than 20/40). Moreover, 1 subject (9.1%) in the CB group had a logMAR BCVA greater than 1.0 (worse than 20/200).

Six months after surgery, logMAR BCVA had improved to 0.10 (0.00–0.20, 20/25) in the CB group and 0.10 (0.10–0.30, 20/25) in the SST group (*P* = .30). Furthermore, 54.5% of subjects in both groups had a logMAR BCVA less than 0.1 (better than 20/25). Additionally, both groups had patients who achieved a logMAR BCVA of 0.0 or lower (better than 20/20; CB group: 36.4%, SST group: 22.7%; *P* = .43).

### 3.4 Corneal thickness

Median preoperative corneal thickness was 614.0 μm (577.5–662.0 μm) and 658.0 μm (606.0–689.0 μm) respectively in the CB and SST groups (*P* = .37). In both groups, corneal thickness increased from preoperative values 7 days after surgery, decreased over the next few weeks, and stabilized between 3 and 6 months after surgery. Six months after surgery, corneal thickness was lower in the CB group (median = 571.0 μm [478.0–592.0 μm]) than in the SST group (median = 576.0 μm [531.0–607.0 μm]). When final corneal thickness was adjusted for follow-up time, corneas in the CB group were significantly thinner than in the SST group (*P* = .02). On average, the reduction from baseline in corneal thickness was 11% in the CB group and 9% in the SST group, a small difference that was not statistically significant (*P* = .79).

### 3.5 Endothelial cell loss

Median ECD before surgery, as evaluated by cell numeration with trypan blue, was 2784 cell/mm^2^ (2612–2995 cell/mm^2^) in the CB group and 2694.5 cell/mm^2^ (2594–2860 cell/mm^2^) in the SST group. Six months after DMEK, more cell loss had occurred in the SST group (1373.5 cell/mm^2^ [566.0–1772.2 cell/mm^2^]) than in the CB group (1005.0 cell/mm^2^ [810.0–1481.0 cell/mm^2^]) and the final ECD was 1403.0 cell/mm^2^ (972.5–2010.7 cell/mm^2^) in the SST group and 1500.0 cell/mm^2^ (1321.5–2049.0 cell/mm^2^) in the CB group. However, this difference was not statistically significant (*P* = .70). The percentage of ECD loss was 41% and 46% in the CB and SST groups, respectively, a small difference that was not statistically significant (*P* = .74). Table 3 summarizes ECD in both donor and recipient tissue.

**Table 3:**
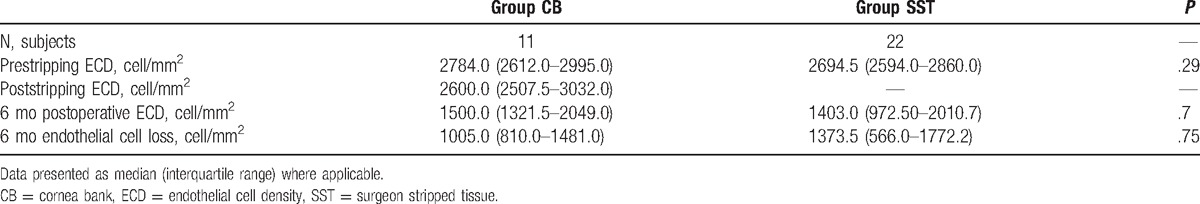
Endothelial density in donor tissue and in subjects following surgery.

### 3.6 Complications

Complications are described in detail in Table 4. Briefly, 3 subjects in each group (CB: 27.3%, SST: 13.6%) developed ocular hypertension (*P* = .37), 1 subject in the CB group (9.1%) and 3 subjects in the SST group (13.6%) developed Irvine–Gass syndrome (*P* > .99), and 2 subjects in the SST group (9.1%) developed pupillary block (0 subjects in CB group, *P* = .54). Tissue stripping failure did not occur at the eye bank, but occurred in 1 case (graft tear) in the OR (4.5%, *P* > .99).

**Table 4:**
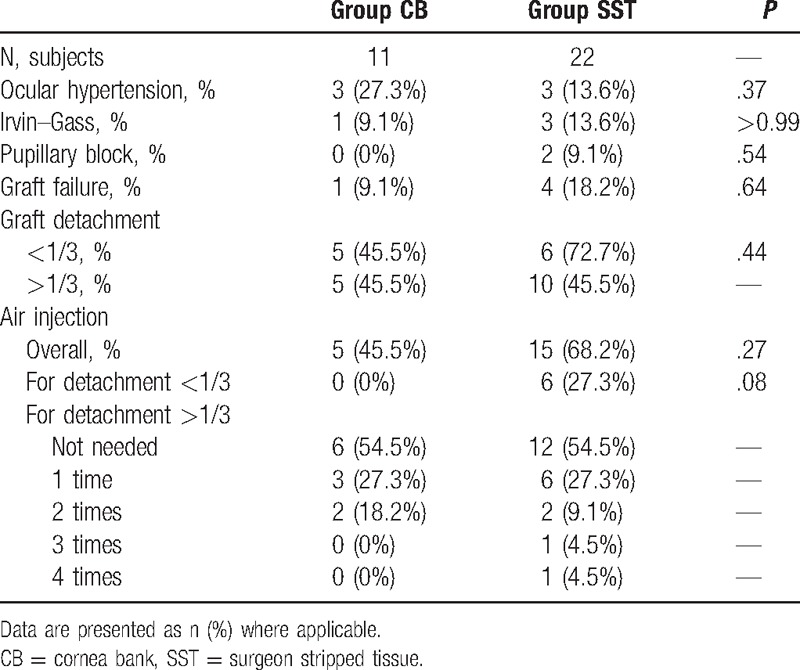
Complications associated with Descemet membrane endothelial keratoplasty in subjects with Fuchs corneal dystrophy.

### 3.7 Incidence of rebubbling

Five subjects (45.5%) in the CB group and 15 subjects (68.2%) in the SST group required rebubbling (*P* = .27). Additionally, 5 subjects (45.5%) in the CB group and 6 subjects (27.3%) in the SST group developed partial detachments (< 1/3, *P* = .43). Interestingly, none of the CB subjects with partial detachments benefited from rebubbling, but all SST patients (6 [27.3%]) did (*P* = .07). Larger detachments (> 1/3) also occurred in 5 CB subjects (45.5%) and 10 (45.5%) SST subjects (*P* > .99).

A single rebubbling was needed in 3 subjects (27.3%) in the CB group and 6 subjects (27.3%) in the SST group. Two rebubblings were needed to pin the graft in 2 subjects (18.2%) in the CB group and 2 subjects (9.1%) in the SST group. In the SST group, 1 subject (4.5%) required 3 rebubblings and 1 subject (4.5%) required 4. These incidences are summarized in Table 4.

### 3.8 Graft failure rates

Only 1 subject (9.1%) had a graft failure in the CB group. This subject did not choose to undergo a second surgery. Significantly more subjects in the SST group experienced graft failure (4 subjects [18.2%]) than in the CB group (*P* = .64). Additionally, 1 subject (4.5%) in the SST group required a transfixing keratoplasty. Graft rejection was not observed in either group (Table 4).

## 4 Discussion

To the best of our knowledge, a comparative study on graft preparation has not yet been published for the DMEK procedure. The current study clearly demonstrates that using CB prestripped grafts in DMEK can result in good clinical outcomes. In this study, a French CB prepared the tissue and we presume that American and Rotterdam CBs would have similar outcomes.

The efficacy of DMEK has been proven and the procedure is now recognized as the best treatment for endothelial dystrophies (e.g., Fuchs dystrophy and bullous keratopathy) and previous penetrating keratoplasty failures.^[23,24]^ Therefore, surgeons have requested that CB technicians be specifically trained in DMEK tissue preparation. The CB staff has been trained, each step in the DMEK tissue preparation process has been validated, quality control techniques (i.e., microbiology and cell count) have been implemented, and DMEK graft tissue stability has been validated using ECD loss (< 7% loss, 3 days following preparation). Additionally, a previous study examined 20 graft preparations and only 1 preparation failure (5%) occurred.^[25]^ Therefore, the Lyon CB should receive the French National Agency for Medicines and Health Products (Agence nationale de sécurité du médicament et des produits de santé—ANSM) authorization to prepare and deliver graft tissue to the OR.

Our 5% stripping failure rate in the SST group matches the 5% tissue preparation failure rate caused by a strong DM-to-stroma adherence that is often present in tissue from young donors.^[26,27]^ In contrast, no tissue preparation failures occurred at the CB. It may be that surgeons will not want to give up preparing the donor button themselves, but this continues the risk of delaying surgery when donor tissue preparation mishaps occur. It should be emphasized to surgeons that using CB prestripped donor tissue eliminates the risk of delay, decreases surgeon stress, shortens procedure time, and eliminates additional OR costs when a second surgical time needs to be scheduled to perform the delayed DMEK.^[15]^

We limited our study sample to Fuchs patients who had not undergone a previous corneal transplant to obtain the most homogeneous population possible. Additionally, Fuchs dystrophy is the most common endothelial dysfunction disorder in which treatment with endothelial transplant leads to the best outcomes.^[23,28]^ Our study had a relatively small number of subjects because we began enrollment a short time after authorization of CB-prepared tissue. However, the aim of the current study was not to test DMEK efficacy, this procedure has already been accepted by the international community, but to observe how patients, surgeons, and the French community may benefit from using CB-prepared donor tissue.

The DMEK procedure is beneficial compared with a full-thickness keratoplasty and DSAEK because of a more rapid visual recovery with comparable endothelial cell loss.^[5,29]^ Our study provides further evidence of desirable visual outcomes with 36.4% and 22.7% of CB and SST subjects, respectively, achieving a BCVA of 20/20 or better 6 months after DMEK. Our results are also in agreement with those of a large cohort study (n = 500 subjects) in which 36% of subjects had a final BCVA of 20/20 or better.^[30]^ Our endothelial cell loss rates were also similar to previously reported values. Our CB-prepared tissue had a 41% endothelial cell loss, which was close to the 37% observed by Rodríguez-Calvo-de-Mora et al,^[30]^ the 30.5% observed by Deng et al,^[31]^ the 32% observed by Tourtas et al,^[29]^ and the 41% observed by Price et al.^[3]^ However, an endothelial cell loss bias cannot be ignored because 2 different methods were used to evaluate ECD before and after surgery. Preoperative ECD was determined in the CB group using trypan blue staining and cell counting under a calibrated reticle. In contrast, postoperative ECD was determined using a specular microscope (SM). The trypan blue method has been reported to overestimate endothelial density when compared with counting apoptotic cells. Unfortunately, counting apoptotic cells is a destructive method and is not an option for examining graft tissue.^[32]^ It should also be noted that automatic SM often underestimate ECD measurements, particularly in edematous corneas.^[29]^ Unfortunately, we could not determine the correlation between the 2 measurement methods because our SM was not usable at the CB.

Endothelial cell loss following tissue stripping was very low in this study (1%). This is an improvement over previously published rates from our CB (3.3%),^[25]^ which likely resulted from the additional daily CB technician training. Endothelial loss between the time of tissue stripping and 6 months after grafting may have been caused by any or all of the following: trephination,^[33]^ graft insertion into the injector,^[34]^ donor tissue injection into the host anterior chamber,^[35]^ the deployment procedure,^[36]^ and postoperative air injection to treat graft detachment.^[37]^ It has also been reported that 2 or more air injections lead to further endothelial cell loss.^[37]^ Therefore, this may explain the higher rate of endothelial cell loss observed here (41% in the CB group, 46% in the SST group) than by Feng et al^[37]^ (26%) because more of our subjects required 2 or more air injections (∼18%) than those in the Feng et al^[37]^ study (7%).

Central corneal thickness (CCT) measurements that had been adjusted for postoperative follow-up time indicated that corneas in the CB group were thinner than corneas in the SST group (*P* = .02). The measured decrease in CCT was 11.6% in the CB group and 8.9% in the SST group (*P* = .79), a decrease that was much lower than that observed in a pachymetry study by Rodríguez-Calvo-de-Mora et al.^[30]^ This large difference may have resulted from the lower preoperative CCT in the CB group than in the Rodríguez-Calvo-de-Mora et al^[30]^ (667 ± 92 μm), Tourtas et al^[29]^ (652 ± 92 μm), and Price et al^[3]^ (656 μm [506–1030 μm]) studies. In contrast, 6 months after DMEK, CCT was slightly higher in our subjects than in the other studies (Rodríguez-Calvo-de-Mora et al^[30]^: 525 ± 46 μm, Tourtas et al^[29]^: 517 ± 45 μm, and Price et al^[3]^: 528 μm [424–678 μm]).

Our air injection rate of 45.5% was relatively high for prestripped corneas. However, it was well within the large range reported in the literature (5.9%–82%) for the DMEK procedure.^[3,13,28]^ A significant association between air injection rates and surgeon experience has already been demonstrated, with rebubblings decreasing from 20% to 4.4% as surgeon experience increased.^[13]^ Tissue storage can also affect air injection rates. Monnereau et al^[14]^ showed that detachment rates were significantly higher with cold storage (34.6%) than with organ culture storage (26.5%). Therefore, our use of organ culture storage medium likely kept detachment rates lower. Feng et al^[20]^ and Heinzelmann et al^[21]^ have both reported their comparative studies on precut tissue using cold storage in DMEK surgery. Precutting was performed by the surgeon the day of surgery or 1 to 2 days before. In Feng study,^[20]^ the preparation 1 to 2 days prior was validated. In donor tissue prepared 2 days ahead of surgery, only 2.8% failure occurred (9.1% in CB), 14% of patients needed air reinjection (45.5% in CB), and the median cell loss at 3 months was 28% (41% at 6 months in CB). Recently, Heinzelmann et al^[21]^ described a higher graft failure rate compared with standard preparation. This can be explained by the use of dextran in the deswelling medium, which is known to be cytotoxic after 2 days of storage. Moreover, the average ECD of precut grafts was 2260 cell/mm^2^ before stripping. In our group, as well as in the Feng et al^[20]^ study, the success of DMEK was obtained with preoperative ECD ≥ 2500 cell/mm^2^.

Our study preliminarily confirms that using a prestripped cornea in DMEK shortens OR times reduces surgeon stress, avoids surgery postponement due to tissue mishaps, and eliminates hospital costs of unproductive OR time. Our results suggest that the highly technical tissue stripping procedure can be successfully performed by trained CB technicians who have worked closely with surgeons. Furthermore, as previously described by Parekh et al,^[38]^ our CB is in current collaboration with surgeons to develop a graft preloading system, which would allow, “reproducibility, reduced surgical time, and reduced tissue wastage, cost, and logistical requirements.” This collaboration is a new advance and challenge for cornea eye banks. It has been shown by others that technique standardization does not improve visual results.^[13,14]^ However, it has other benefits to both patients and hospitals and allows DMEK to be more accessible.^[39]^

In 2006, Hsiue et al^[40]^ and in 2007 Lai et al^[41]^ described a novel cell sheet-based therapy to avoid perforating and posterior keratoplasties. A transplantable human corneal endothelial cell (HCEnC) monolayer was obtained in vitro with a cultivated cell sheet through external temperature modulation of thermoresponsive culture substrates. This bioengineered, human corneal endothelial cell transplantation using cultivated cells demonstrated a promising outcome with rabbit corneas. After the removal of the corneal endothelium, a 7-mm diameter gelatin disc with HCEnCs was implanted in the anterior chamber through a 7.5 mm sclerocorneal incision, which was sutured with 3 stitches. In the HCEnCs sheet group, the CCT increased by 892.7 ± 52.4 μm, then decreased to 504.4 ± 24.7 μm (*P* < .001) at 6 months postgrafting. Corneal transparency was restored within 2 weeks postoperative, and histologic examination showed an intact cell monolayer at 6 months. In the control group without the HCEnCs sheet, the CCT remained at a high level during the experimental period. This technique could overcome the limitation of the shortage of donor corneas with faster rehabilitation and less tissue trauma, but it has not yet been tested in humans.

Our study had several limitations. These include its small sample size, short follow-up period (6 months), and retrospective design. Larger, multicenter, long-term, prospective studies are needed to confirm our findings and to further examine clinical outcomes of DMEK procedures using CB-prepared tissue. In summary, our study shows that CB stripping of donor tissue for DMEK has similar anatomic and functional outcomes to procedures performed with SST when used to treat Fuchs dystrophy.
